# Relationship between Oral Bacterial Count and Postoperative Complications among Patients with Cardiovascular Disease Treated by Surgery: A Retrospective Cohort Study

**DOI:** 10.3390/healthcare9070850

**Published:** 2021-07-05

**Authors:** Rie Osako, Yuhei Matsuda, Chieko Itohara, Yuka Sukegawa-Takahashi, Shintaro Sukegawa, Satoe Okuma, Yoshihiko Furuki, Takahiro Kanno

**Affiliations:** 1 Department of Oral and Maxillofacial Surgery, Faculty of Medicine & Oral Care Center, Shimane University Hospital, Izumo 693-8501, Japan; r.osako@med.shimane-u.ac.jp (R.O.); yuhei@med.shimane-u.ac.jp (Y.M.); ioa2thr@med.shimane-u.ac.jp (C.I.); okuma125@med.shimane-u.ac.jp (S.O.); 2 Department of Oral and Maxillofacial Surgery, Kagawa Prefectural Central Hospital, Takamatsu 760-8557, Japan; yuka611225@gmail.com (Y.S.-T.); gouwan19@gmail.com (S.S.); furukiy@ma.pikara.ne.jp (Y.F.)

**Keywords:** cardiovascular disease, oral care, oral bacterial count, perioperative oral management, postoperative complication, retrospective cohort study, surgery

## Abstract

In this retrospective observational study, we evaluated the relationship between perioperative oral bacterial counts and postoperative complications in cardiovascular disease (CVD) patients. From April 2012 to December 2018, all patients scheduled for surgery received perioperative oral management (POM) by oral specialists at a single center. Tongue dorsum bacterial counts were measured on the pre-hospitalization day, preoperatively, and postoperatively. Background data were collected retrospectively. Among the 470 consecutive patients, the postoperative complication incidence rate was 10.4% (pericardial fluid storage, *n* = 21; postoperative pneumonia, *n* = 13; surgical site infection, *n* = 9; mediastinitis, *n* = 2; and seroma, postoperative infective endocarditis, lung torsion, and pericardial effusion, *n* = 1 each). Oral bacterial counts were significantly higher in the pre-hospitalization than in the pre- and postoperative samples (*p* < 0.05). Sex, cerebrovascular disease, and operation time differed significantly between complications and no-complications groups (*p* < 0.05). Multivariate analysis with propensity score adjustment showed a significant association between postoperative oral bacterial count and postoperative complications (odds ratio 1.26; 95% confidence interval, 1.00–1.60; *p* = 0.05). Since the development of cardiovascular complications is a multifactorial process, the present study cannot show that POM reduces complications but indicates POM may prevent complications in CVD patients.

## 1. Introduction

Microbiota collected from the oral cavity generally include 50% *Streptococcus* spp., 32% *Staphylococcus* spp., 6% Gram-negative bacteria, and 1% fungi [1]. It has been reported that oral bacteria, particularly anaerobic Gram-negative bacteria, which are a source of periodontal disease, cause not only surgical site infection, but also remote infection, such as infective endocarditis [2]. According to a large Japanese survey conducted between 2000 and 2001, dental treatment was the most common cause of infective endocarditis, excluding unknown causes [1]. Additionally, based on the results of this study, the first guideline for the prevention and treatment of infective endocarditis in Japan was established in 2003, and it was strongly recommended that "the use of antimicrobial agents is necessary in the dental treatment of patients at risk of cardiac disease”, and oral management and care should be well mentioned during the treatment of cardiovascular disease (CVD) [3].

In recent years, it has been reported that bacteria-related postoperative complications encountered in the treatment of cardiovascular diseases, including valve surgery, result in a delayed recovery period and increased postoperative mortality [4]. Dyslipidemia is thought to be the main cause, but it has become clear that dental caries and periodontal bacteria are etiological factors in a variety of cardiovascular diseases [5]. In 2015, Oliveira et al. successfully detected oral bacteria in the heart valves of patients with cardiovascular diseases [6]. It has been suggested that these oral bacteria may cause adverse immune reactions after cardiovascular surgery, not only via local infection, but also by releasing inflammatory mediators [7]. In addition, a retrospective case‒control study of 223 patients with heart valve disease in 2020 concluded that the use of oral care plays an important role in decreasing postoperative inflammatory complications [8]. However, to date, most of the methods used in many clinical studies have examined the relationship between the implementation of perioperative oral management and postoperative complications, whereas no studies have directly examined the relationship between bacterial counts and postoperative complications. In fact, previous studies conducted by our research team also showed that oral status before surgery in patients with lung cancer was associated with postoperative fever [9].

On the other hand, while oral bacterial counts in the perioperative period have received much attention, oral status, including daily oral bacterial counts, may also influence postoperative complications [10]. It is possible that many patients with cardiovascular disease routinely suffer from oral diseases, such as periodontal disease. Okuda et al. reported that periodontal bacteria were detected in esophageal aneurysms and diseased cardiac coronary arteries, and pointed out that oral bacteria may be involved in inducing atherosclerosis [11]. This phenomenon may be related to the suggestion that oral bacteria with platelet-aggregating ability accelerate atherosclerosis [11]. Tonetti et al. conducted a parallel-group, single-blind, randomized, controlled trial comparing 120 patients with severe periodontitis in a general periodontal treatment group and an advanced periodontal treatment group, and reported that advanced periodontal treatment improved the condition of vascular endothelial cells in the arterial wall [12]. Therefore, patients with cardiovascular disease may have more oral disease as a background factor than healthy individuals. In other words, it could be hypothesized that the high prevalence of periodontal disease in patients with cardiovascular disease may be a background factor that exacerbates the incidence of postoperative complications in cardiovascular treatment. To test this hypothesis, it is necessary to configure study designs that consider the number of oral bacteria before perioperative management is initiated.

In recent years, instead of counting colony-forming units (CFUs), which are the gold-standard for bacterial counting, the dielectrophoretic impedance measurement (DEPIM) method has been used to measure the number of oral bacteria easily [9]. DEPIM is a measurement method in which bacteria in a liquid are collected by electrodes using dielectrophoresis, and the change in impedance is measured and converted into the concentration of bacteria (CFU/mL) in 1 mL of the specimen. Use of this apparatus for measuring oral bacterial count has revealed that more severe periodontal disease progression indirectly reflected worse oral hygiene. Since the measurement is performed on a specimen obtained by swabbing the center of the dorsum of the individual’s tongue, it has gained attention as a non-invasive and low-cost method for measuring the number of bacteria [13].

Thus, based on the hypothesis that the prevalence of oral bacteria count in patients with cardiovascular disease may be a background factor that exacerbates the incidence of postoperative complications in cardiovascular treatment, we evaluated the relationship between perioperative oral bacterial counts and postoperative complications in patients with cardiovascular disease using DEPIM.

## 2. Materials and Methods

This study was conducted in accordance with the STROBE statement.

### 2.1. General Perioperative Oral Management in Kagawa Prefectural Central Hospital

Initiated by the newly established “perioperative oral function management” by the revision of dental fees in April 2012, the dentists, oral surgeons, and dental hygienists of Kagawa Prefectural Central Hospital introduced a system to carry out POM for all patients undergoing surgery. Specifically, oral evaluation (interview and evaluation of teeth, periodontal tissue, mucosa, and dentures) was performed before admission, and oral evaluation, oral cleaning, and oral hygiene instruction were performed the day before surgery, and the same POM was performed the day before discharge. Intraoral bacterial counters were routinely used. This was done three times for each patient as an outcome of POM and for feedback.

### 2.2. Recruitment of Research Subjects and Data Collection Methods

**Figure 1:**
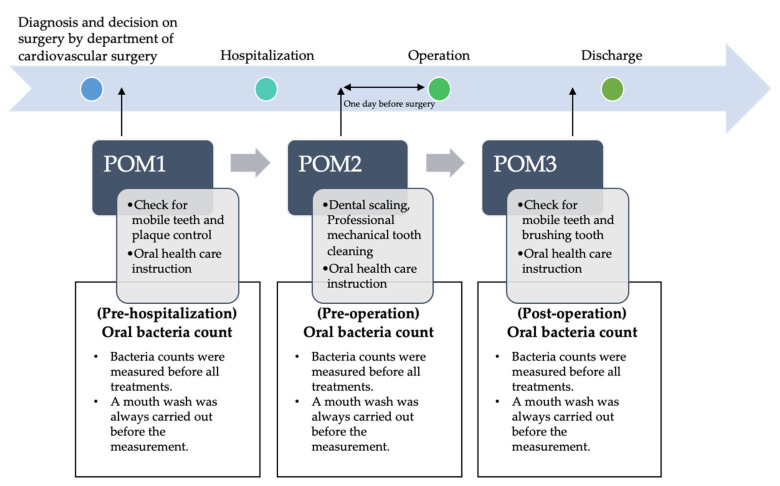
Perioperative oral management (POM) flow and interventions and timing of the oral bacterial count.

The study design of this study is a retrospective, single-center cohort study with risk factors for postoperative complications as the primary outcome. All patients undergoing treatment for cardiovascular disease at Kagawa Prefectural Central Hospital had a system in place for receiving POM at the oral care center during the perioperative period, including before admission, as described above. The study subjects were those who visited the oral care center for the prevention of perioperative complications related to the oral cavity between April 2012 and December 2018 at the Kagawa Prefectural Central Hospital (Kagawa, Japan). All study subjects were then provided with informed consent and, after obtaining consent, underwent POM after three measurements of the oral bacterial count using an oral bacterial counter. The flow diagram of POM is shown in Figure 1. All patients were advised to undergo regular oral and dental care on a total of three occasions throughout the perioperative period.

This study was conducted after obtaining approval from the Medical Ethics Committee of Shimane University (No. 4041) and the Ethics Committee of Kagawa Prefectural Central Hospital (No. 878). Patients who were unable to give informed consent for POM and those who were unable to have their oral bacterial count measured a total of three times (as part of the regular POM course, on the day they visited the oral care center before admission, the day before surgery, and the day before discharge) were excluded from the study and not included in the analysis.

### 2.3. Study Variables

The following data were collected via sequential sampling method: patient characteristics (age, sex (male/female), body mass index (kg/m^2^)), performance status, Brinkman index, primary disease (angina, myocardial infarction, aortic aneurysm, aortic dissection, valvular disease, cardiomyopathy, atrial septal defect, heart tumor, heart failure, arrhythmia, and others), medical history (diabetes mellitus, cerebrovascular disease, cancer, dementia, and rheumatic disease), number of teeth, denture use, involvement of a home dentist, operation time (minutes), preoperative white blood cell counts (10^3^/μL), preoperative serum albumin values g/dL], and duration of hospitalization [days]).

A home dentist was defined as a patient’s regular visit to a dental clinic within the last year. For operative time, information (minutes) was extracted from electronic medical records for operations performed by a single surgical team at the Department of Cardiovascular Surgery, Kagawa Prefectural Central Hospital. Therefore, selection bias may exist because this study was limited to patients who were able to understand the purpose of the study.

### 2.4. Oral Bacterial Count

**Figure 2:**
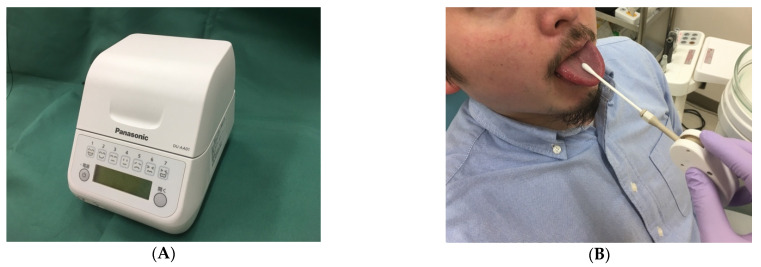
Quick and easy oral bacteria counting device (Panasonic Healthcare, Tokyo, Japan). (**A**) Frontal view of the oral bacteria counter (**B**) Specimen being collected from the middle of the dorsal tongue using an applicator.

Figure 2A shows the oral bacterial count on the central dorsal surface of the tongue and the bacterial detection device (Panasonic Healthcare, Tokyo, Japan), with a clinically experienced dental hygienist operating the device before admission, preoperatively, and postoperatively, based on the procedure reported in a previous study by Itohara et al. [9]. In order to ensure reproducibility and objectivity, the measurements were performed according to a pre-specified procedure to minimize measurement errors. Specifically, calibration was performed by impregnating the sample with 50 mL of water before every measurement. To standardize the sampling, the sampling site was kept constant and samples were collected using a universal applicator. Furthermore, the examiners were adequately trained to calibrate and minimize any deviations in sample collection (Figure 2B). The results of the oral bacterial counts were automatically graded by the instrument into the following categories (CFU/mL): <10^6.5^ (level 1); ≥10^6.5^ to <10^7^ (level 2); ≥10^7^ to <10^7.5^ (level 3); ≥10^7.5^ to <10^8^ (level 4); ≥10^8^ to <10^8.5^ (level 5); ≥10^8.5^ to <10^9^ (level 6); and ≥10^9^ (level 7).

### 2.5. Study Outcomes

Based on the Japan Clinical Oncology Group (JCOG) postoperative complication criteria (Clavien-Dindo classification version 2.0), the following complications were considered as postoperative complications: pericardial fluid storage, postoperative pneumonia, surgical site infection, mediastinitis, seroma, postoperative infective endocarditis, lung torsion, and pericardial effusion [14].

### 2.6. Statistical Analyses

All statistical analyses were carried out using SPSS version 26.0 software (IBM Japan, Tokyo, Japan). The Friedman test was used for comparisons at each time point (pre-admission, preoperative and postoperative), followed by Bonferroni’s multiple comparison test. The group comparison of background data was performed using Mann‒Whitney’s U-test and the chi-squared test, depending on the type of variable. Finally, to adjust for confounding factors between the two groups, propensity scores were calculated, and confounding adjustment was performed using the inverse probability of treatment weighting (IPTW). Odds ratios at each time point were calculated using generalized estimating equations. Statistical significance was set at *p* < 0.05. In addition, for the completion of missing values, the multiple substitution method with the logistic imputation method was adopted, assuming that the missing data were missing completely at random.

## 3. Results

### 3.1. Patients Characteristic

A total of 470 consecutive patients (301 men and 169 women) were enrolled in the study. Their characteristics are shown in Table 1. Patients tended to be older but were mostly normal weight. Most patients had a performance status (PS) of 0. The primary diseases included angina, myocardial infarction, aortic aneurysm, aortic dissection, valvular disease, cardiomyopathy, atrial septal defect, heart failure, arrhythmia, and other conditions. Almost one-fifth of patients had diabetes mellitus, while others had cerebrovascular disease, cancer, dementia, and rheumatoid disease. The median number of teeth was 17.0. Almost half of the patients used dentures. Three-quarters of patients used a home dentist. The median oral bacterial count was the same at pre-hospitalization, at preoperation, and at postoperation.

### 3.2. Longitudinal Change in Oral Bacterial Count

**Figure 3:**
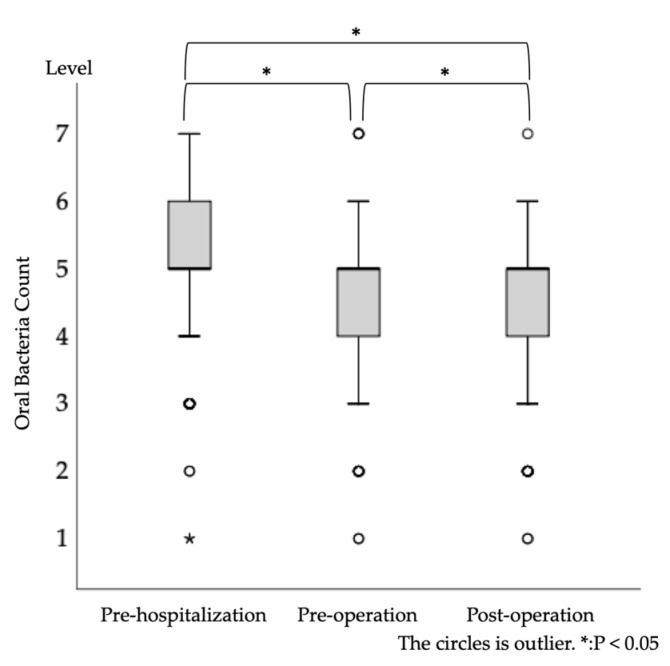
Longitudinal changes in oral bacterial counts (at pre-hospitalization, preoperation, and postoperation). One-way analysis of variance revealed statistically significant differences.

The results of the Friedman test are shown in Figure 3. Oral bacterial count levels were significantly different (*p* < 0.001) at each time point (pre-admission, preoperative and postoperative). In addition, Bonferroni’s multiple comparison test showed significant differences between all groups (*p* < 0.05), between pre-hospitalization and postoperation, and between pre- and postoperation (all *p* < 0.001).

### 3.3. Number of Patients with Postoperative Complications

In total, 49 postoperative complications occurred in this study, and their characteristics are shown in Table 2. The types of complications included pericardial fluid storage, postoperative pneumonia, surgical site infection, mediastinitis, seroma, postoperative infective endocarditis, lung torsion, and pericardial effusion.

### 3.4. Between-Group Comparison of Baseline Background Factors

Table 3 summarizes the results of complication and non-complication group comparisons that investigated the potential risk factors for postoperative complications. In the total data, there were significant differences between the non-complication and complication groups in terms of sex, presence of cerebrovascular disease, and operation time (*p* < 0.05), but no significant differences in the other characteristics.

### 3.5. Propensity Score Analysis of the Association between the Development of Postoperative Complications and Oral Bacterial Count

Table 4 summarizes the results of the propensity score analysis of the association between the development of postoperative complications and oral bacterial count. There were significant correlations between postoperative complications and oral bacterial count at postoperation (odds ratio: 1.26 (95% confidence interval: 1.00–1.60); *p* = 0.049). There were no significant correlations between postoperative complications and pre‒hospitalization and preoperative oral bacterial counts.

## 4. Discussion

In this retrospective observational study, we evaluated the relationship between perioperative oral bacterial counts and postoperative complications in patients with cardiovascular disease. Oral bacterial counts were significantly higher pre-hospitalization than pre- and postoperatively (*p* < 0.05). Sex, cerebrovascular disease, and operation time differed significantly between complications and no-complications groups (*p* < 0.05). Propensity score-adjusted multivariate analysis showed that postoperative oral bacterial count was significantly associated with postoperative complications (odds ratio 1.26; 95% CI, 1.00–1.60; *p* = 0.05). Thus, POM can reduce oral bacterial counts, which could be a risk factor for postoperative complications. Appropriate POM is essential for preventing complications and may play an important role in the perioperative management of patients with cardiovascular diseases.

The subjects of this study were a population of Japanese patients with cardiovascular disease in terms of age, sex, BMI, smoking status, and operation time [8]. According to World Health Organization mortality statistics, Japan has the lowest mortality rate for cardiovascular disease among developed countries. In surveys conducted in various countries, males were reported to have a higher mortality rate for cardiovascular disease than females, and the results of our study showed a similar trend [15]. In the Honolulu Heart Study, which examined Japanese-American men, smoking and impaired glucose tolerance were cited as risk factors, which is consistent with our descriptive statistics [16]. In our study, 22.1% of patients had diabetes mellitus, which is not necessarily a risk factor in major epidemiological studies in Japan, although it has been reported as a significant risk factor in the American population [17]. In terms of oral status, compared to our previous data on perioperative lung cancer patients, these patients had fewer teeth and a higher rate of denture use, which may have resulted in a population with worse oral status as compared to the general healthy population [9]. This may be because diabetes is reportedly a risk factor not only for heart disease, but also for periodontal disease and its interacting factors [18]. Therefore, our descriptive statistics suggest that the population of this study is highly generalizable to Japanese patients with cardiovascular disease overall. In addition, they are also a population with poor oral status, which may predispose them to perioperative oral-related complications.

In our between-group comparisons, sex, cerebrovascular disease, and operation time were associated with the development of complications. Our results are in line with this, as cerebrovascular disease has been identified as a risk factor for postoperative complications [19]. In addition, our results are consistent with the fact that prolonged operation time is generally a risk factor for postoperative complications [20]. However, we could not find any reports indicating that male patients with cardiovascular disease were more likely to have postoperative complications. Rather, a review by Stoberock et al. showed that women have a higher incidence of complications and longer hospital stays than men [21]. This is thought to be due to the generally older age of women at the time of diagnosis and treatment of cardiovascular disease, as well as the presence of genetic, hormonal, anatomical, biological, and socio-cultural differences.

In the propensity score analysis, the postoperative oral bacterial count was significantly associated with the development of postoperative complications. The most significant finding of this study was the identification of not only an association between oral bacterial counts and postoperative complications, but also a clear time frame in which oral bacterial counts were associated with postoperative complications. In our study, a total of eight different postoperative complications were observed. To the best of our knowledge, no previous studies have shown an association between oral bacterial count and pericardial fluid storage, mediastinitis, seroma, lung torsion, and pericardial effusion. The relationship between postoperative pneumonia and a disease condition with accumulation of oral bacteria retention is widely known, and oral bacteria have been reported to cause postoperative pneumonia in patients undergoing brain, esophageal, respiratory, and other surgeries [22,23,24]. Therefore, it is not unusual that similar results were obtained in patients with cardiovascular disease. Oral bacteria have been reported to cause local infection in oral cancer surgery [25]. In addition, oral bacteria can cause infective endocarditis as a remote infection [26]. The present study also suggested that the number of oral bacteria may be related to surgical site infection as a remote infection. It was also suggested that para-inflammation caused by inflammatory cytokines in the bloodstream pathway of periodontitis may have been a factor in the development of complications [7].

In this study, postoperative oral bacterial counts were associated with complications, suggesting the need for thorough preoperative dental plaque and tartar removal and cleaning guidance by dentists and dental hygienists, as well as continuous and intensive oral care in the intensive care unit after surgery to continue to reduce oral bacterial counts.

Future studies are needed to identify strains associated with postoperative oral bacterial counts and the incidence of postoperative complications in patients with cardiovascular disease, and to explore cutoff values for oral bacterial abundance in perioperative management and care. Recently, a device to measure the amount of *Porphyromonas gingivalis*, one of the main components of the red complex, by polymerase chain reaction tests at the chairside was developed [27], and it is expected to be applied in clinical research.

This study has seven limitations: First, the detailed classification of each of the cardiovascular diseases was not considered or analyzed in this preliminary cohort study. Thus, patients were not sub-grouped, but were analyzed together as a group with “cardiovascular diseases,” so that disease-specific characteristics cannot be determined. Second, cholesterol and blood pressure were not considered as background factors in the patients. These three factors, including smoking, have been pointed out in many studies as the three major risk factors for cardiovascular disease and should have been considered in the analysis [28]. Third, the oral bacterial counts handled in this study were not measured by subdividing the strains such as Socransky red complex, but rather were measured by using the total number of bacteria in the oral cavity as a surrogate value, making it difficult to identify the strains that directly caused the complications. Fourth, data on the status of medication were not collected; hence, it is possible that the drugs used affected the results. Fifth, the oral care protocol used in this study may be difficult to adapt for patients living in rural areas due to time constraints. Sixth, the gold-standard periodontal examination and assessment of gingival bleeding were not included in the analysis because no data were collected in this study. Seventh, since no sensitivity analysis was conducted on the imputation of missing values, the accuracy of the results is unreliable, but the missing value assignment was used for only three items, and the number of missing values was very small. The maximum missing value was 4.9% (hospital duration), and since this item was not used in the multivariate analysis, its impact on the main results is considered to be low.

## 5. Conclusions

The study showed that POM can continuously reduce the level of oral bacteria in patients with cardiovascular disease, that the postoperative oral bacteria count is the only risk factor for postoperative complications, and that continuous intervention by dentists/oral surgeons and dental hygienists before hospital admission is essential to prevent complications. Since the development of cardiovascular complications is a multifactorial process, this study cannot show that oral care reduces complications but indicates that continuous intervention by dentists/oral surgeons and dental hygienists before hospital admission may be essential to prevent complications. Therefore, POM may play an important role in the perioperative management of patients with cardiovascular disease.

## Figures and Tables

**Table 1:** Patient demographics and characteristics (*N* = 470).

Characteristics	Category	*N* (%) or Median (IQR)
Age		76.0 (69.0–81.0)
Sex	Male	301 (64.0)
	Female	169 (36.0)
Body mass index		21.9 (19.6–24.6)
Performance status	0	438 (93.2)
	1	12 (2.6)
	2	13 (2.8)
	3	4 (0.9)
	4	3 (0.6)
Brinkman Index		0.0 (0.0–800.0)
Cardiovascular disease	Angina	42 (8.9)
	Myocardial infarction	7 (1.5)
	Aortic aneurysm	162 (34.5)
	Aortic dissection	3 (0.6)
	Valvular disease	212 (45.1)
	Cardiomyopathy	1 (0.2)
	Atrial septal defect	4 (0.9)
	Heart tumor	5 (1.1)
	Heart failure	2 (0.4)
	Arrhythmia	6 (1.3)
	Arteriosclerosis obliterans	4 (0.9)
	Popliteal aneurysm	2 (0.4)
	Iliac artery aneurysm	12 (2.6)
	Myocarditis	1 (0.2)
	Internal carotid artery stenosis	1 (0.2)
	Aortic dilation	2 (0.4)
	Ventricular aneurysm	1 (0.2)
	Rurish syndrome	1 (0.2)
	Cardiogenic cerebral embolism	1 (0.2)
	Infective endocarditis	1 (0.2)
Medical history	Diabetes mellitus	104 (22.1)
	Cerebrovascular disease	61 (13.0)
	Cancer	85 (18.1)
	Dementia	8 (1.7)
	Rheumatoid	6 (1.3)
Number of teeth		17.0 (5.0–25.0)
Denture	Yes	233 (49.6)
Home dentist	Yes	359 (76.4)
Oral bacterial count at pre-hospitalization	(10^6^ CFU/mL)	26.8 (14.2–47.1)
	Level	5.0 (5.0–6.0)
Oral bacterial count at preoperation	(10^6^ CFU/mL)	15.8 (7.4–31.2)
	Level	5.0 (4.0–5.0)
Oral bacterial count at postoperation	(10^6^ CFU/mL)	13.2 (6.4–23.5)
	Level	5.0 (4.0–5.0)
Operation time	(minutes)	305.5 (204.8–382.0)
White blood cell count at preoperation	10^3^/μL	6.0 (4.9–7.3)
Serum albumin value at preoperation	g/dL	4.0 (3.6–4.3)
Hospital duration	Day	17.0 (14.0–23.0)

CFU: colony-forming unit, IQR: interquartile range, SD: standard deviation.

**Table 2:** Classification and number of patients with postoperative complications based on Japanese Clinical Oncology Group postoperative complication criteria (Clavien‒Dindo classification).

Type of Complication	*N* (%)
Pericardial fluid storage	21 (42.9)
Postoperative pneumonia	13 (26.5)
Surgical site infection	9 (18.4)
Mediastinitis	2 (4.1)
Seroma	1 (2.0)
Postoperative infective endocarditis	1 (2.0)
Lung torsion	1 (2.0)
Pericardial effusion	1 (2.0)

**Table 3:** Between-group comparison of baseline background factors for the presence of complications.

Variables	Category	*N* (%) or Median (IQR)		*p*-Value
		Non-Complication (*N* = 421)	Complication (*N* = 49)	
Age		76.0 (69.0–81.0)	73.0 (68.0–78.0)	0.11 ^a^
Sex	Male	263 (62.5)	38 (77.6)	0.04 ^b,^*
	Female	158 (37.5)	11 (22.4)	
Body mass index		22.0 (19.8–24.5)	21.2 (18.5–24.9)	0.25 ^a^
Performance status	0	391 (92.9)	47 (95.9)	0.44 ^a^
	1	12 (2.9)	0 (0)	
	2	12 (2.9)	1 (2.0)	
	3	3 (0.7)	1 (2.0)	
	4	3 (0.7)	0 (0)	
Brinkman Index		0.0 (0.0–770.0)	450.0 (0.0–900.0)	0.05 ^a^
Cardiovascular disease	Angina	40 (9.5)	2 (4.1)	–
	Myocardial infarction	6 (1.4)	1 (2.0)	
	Aortic aneurysm	145 (34.4)	17 (34.7)	
	Aortic dissection	3 (0.7)	0 (0)	
	Valvular disease	190 (45.1)	22 (44.9)	
	Cardiomyopathy	1 (0.2)	0 (0)	
	Atrial septal defect	4 (1.0)	0 (0)	
	Heart tumor	4 (1.0)	1 (2.0)	
	Heart failure	1 (0.2)	1 (2.0)	
	Arrhythmia	6 (1.4)	0 (0)	
	Arteriosclerosis obliterans	4 (1.0)	0 (0)	
	Popliteal aneurysm	1 (0.2)	1 (2.0)	
	Iliac artery aneurysm	12 (2.9)	0 (0)	
	Myocarditis	0 (0)	1 (2.0)	
	Internal carotid artery stenosis	1 (0.2)	0 (0)	
	Aortic dilation	2 (0.5)	0 (0)	
	Ventricular aneurysm	0 (0)	1 (2.0)	
	Rurish syndrome	1 (0.2)	0 (0)	
	Cardiogenic cerebral embolism	0 (0)	1 (2.0)	
	Infective endocarditis	0 (0)	1 (2.0)	
Medical history	Diabetes mellitus	92 (21.9)	12 (24.5)	0.72 ^b^
	Cerebrovascular disease	50 (11.9)	11 (22.4)	0.04 ^b,^*
	Cancer	73 (17.3)	12 (24.5)	0.24 ^b^
	Dementia	7 (1.7)	1 (2.0)	0.59 ^b^
	Rheumatoid	6 (1.4)	0 (0)	1.00 ^b^
Number of teeth		17.0 (6.0–25.0)	17.0 (2.0–26.0)	0.91 ^a^
Denture	Yes	207 (49.2)	26 (53.1)	0.65 ^b^
Home dentist	Yes	316 (75.1)	43 (87.8)	0.05 ^b^
Oral bacterial count at pre-hospitalization	Level	5.0 (5.0–6.0)	5.0 (5.0–6.0)	0.65 ^a^
Oral bacterial count at preoperation	Level	5.0 (4.0–5.0)	5.0 (4.0–6.0)	0.28 ^a^
Oral bacterial count at postoperation	Level	5.0 (4.0–5.0)	5.0 (4.0–5.0)	0.08 ^a^
Operation time	(minutes)	297.0 (196.0–368.0)	379.0 (311.0–467.0)	<0.01 ^a,^*
White blood cell count at preoperation	10^3^/μL	6.0 (4.9–7.4)	6.0 (4.9–7.2)	0.95 ^a^
Serum albumin value at preoperation	g/dL	4.0 (3.6–4.3)	4.0 (3.5–4.3)	1.00 ^a^

CFU, colony-forming unit; IQR, interquartile range; SD, standard deviation; ^a^ Mann‒Whitney U-test; ^b^ chi-squared test; *: *p* < 0.05.

**Table 4:** Odds ratios with propensity score analysis of the association between the development of postoperative complications and the number of oral bacteria at each time point.

Explanatory Variable		Odds Ratio (CI)	*p*‒Values
Oral bacterial count at pre-hospitalization	Level	0.90 (0.69–1.17)	0.43
Oral bacterial count at preoperation	Level	1.14 (0.84–1.56)	0.40
Oral bacterial count at postoperation	Level	1.26 (1.00–1.60)	0.05 *

CI, 95% confidence interval; * *p* < 0.05. We used age, sex, performance status, Brinkman index, cerebrovascular disease, number of teeth, denture, operation time, preoperative white blood cell count, and preoperative serum albumin values as adjusted confounders for calculating the propensity score.

## Data Availability

The data of this study are not available.
